# The Impact of Diabetic Retinopathy on the Choriocapillaris in Neovascular AMD

**DOI:** 10.1167/iovs.64.14.32

**Published:** 2023-11-21

**Authors:** Pasquale Viggiano, Alexandra Miere, Enrico Borrelli, Giacomo Boscia, Maria Oliva Grassi, Eric H. Souied, Giovanni Alessio, Francesco Boscia

**Affiliations:** 1 Department of Translational Biomedicine Neuroscience, University of Bari “Aldo Moro,” Bari, Italy; 2 Department of Ophthalmology, Centre Hospitalier Intercommunal de Créteil, Université Paris Est, Créteil, France; 3 Ophthalmology Department, San Raffaele University Hospital, Milan, Italy

**Keywords:** nAMD, diabetic retinopathy, OCTA, macular neovascularization

## Abstract

**Purpose:**

To investigate the impact of diabetic retinopathy (DR) on morphological choriocapillaris (CC) modifications in eyes with type 1 macular neovascularization (MNV) secondary to AMD using optical coherence tomography angiography (OCTA).

**Methods:**

Eyes with AMD-related type 1 MNV with and without DR were prospectively included. We performed 3 × 3 mm OCTA scans at two visits: before the loading phase of intravitreal injections of aflibercept (T1) and 1 month after the last injection (T2). OCTA En face flow images of the CC were analyzed for flow deficit percentage (FD%), FD average area and FD number in a 500-µm-wide ring surrounding the dark halo (DH) around type 1 MNV.

**Results:**

A total of 65 eyes, out of which 30 eyes had mild DR, were included. In the group without diabetes, there was a gradual reduction in FD% in the CC ring around the DH after antiangiogenic therapy, indicating reperfusion of the CC (*P* = 0.003). However, in the DR group, there were no significant changes in CC parameters between the two study visits. Specifically, the FD% in the CC ring around the DH did not show a significant reduction at T2 compared with T1 values (*P* > 0.05). Furthermore, the comparison of the variation in FD% between the two groups was statistically significant. The nondiabetic group exhibited a gradual CC reperfusion after the loading phase of aflibercept, whereas the diabetic eyes did not show significant changes (*P* = 0.029).

**Conclusions:**

The CC surrounding the DH associated to type 1 MNV exhibited greater hypoperfusion in diabetic eyes compared with eyes without diabetes, both before starting therapy and after the loading phase. Hence, DR may be a potential risk factor in the development and progression of late-stage AMD and may also influence the response to antiangiogenic therapy.

AMD is the leading cause of blindness among the elderly population and its incidence and prevalence are expected to increase.^1^ The pathogenesis of AMD is complex and involves various environmental and genetic factors such as age, smoking, cardiovascular disease, and diabetic angiopathy.^2^ Moreover, diabetes mellitus (DM) has been suggested as a possible risk factor for AMD, although the relationship between the two conditions is still not fully understood,^3^ and the results in current literature are discordant.^4^

Diabetic retinopathy (DR), one of the most frequent retinal vascular diseases, has a relatively high prevalence in DM patients.^3^ Although AMD is fundamentally considered as an outer retinal disease, affecting the RPE–Bruch Membrane–choriocapillaris (CC) complex, and DR is considered a fundamentally inner retinal disease, recent studies have shown that inner layers are also affected from early AMD stages^4^^,^^5^ and, vice versa, the CC is impaired in DM eyes, even in the absence of DR.^6^

Conventional imaging methods in both AMD and DR, such as fluorescein angiography and indocyanine green angiography, have been used to visualize the retinal and choroidal vascular changes.^7^ Nevertheless, neither fluorescein angiography nor indocyanine green angiography are depth resolved and, therefore, are unable to distinguish the thin capillaries of the CC.^8^ Optical coherence tomography angiography (OCTA), an OCT-derived imaging technique, has emerged as a valuable tool for quantifying flow changes in the retina and CC in various chorioretinal diseases and in response to intravitreal treatment.^9^^–^^11^

The CC and the inner choroid are pivotal in the pathophysiological mechanisms of AMD,^12^ because they serve as sources of nourishment and oxygen supply to the RPE.^13^ Hence, a reduction in CC perfusion may contribute to retinal degeneration. For instance, recent advancements in OCTA have shown reduced CC flow in intermediate AMD (iAMD), particularly in areas surrounding drusen and reticular pseudodrusen. This impairment is also observed in late-stage AMD, characterized by macular neovascularization (MNV) and geographic atrophy. Notably, it has been proposed that the most significant CC hypoperfusion occurs in the vicinity of MNV, albeit requiring signal compensation and image averaging for reliable quantitative results.^14^^,^^15^ Using a 200-µm ring referred to as the "halo" zone, Treister et al.^16^ illustrated a notably higher degree of CC nonperfusion adjacent to all MNV lesions.^15^^,^^17^^–^^20^

Interestingly, CC ischemia has been observed in both type 1 and type 2 DM, even in the absence of clinical signs of DR, compared with healthy eyes.^21^ Subsequently, the quantification of flow deficits (FDs) in the CC has been proposed as a novel biomarker for predicting the onset and progression of DR.^6^^,^^22^

Considering the potential influence of DM and DR on CC flow in AMD eyes, we aimed to assess whether the presence of diabetes and DR could affect the distribution of CC flow surrounding type 1 MNV before and after anti-VEGF therapy loading phase.

## Methods

### Study Participants

This prospective cohort study was conducted in the retina department between August 2020 and September 2022. Ethical considerations were followed according to the principles outlined in the Declaration of Helsinki, and the study protocol was approved by the institutional review board of the Department of Translational Biomedicine Neuroscience at the University of Bari “Aldo Moro.” Written informed consent was obtained from all participating patients prior to their inclusion in the study.

A total of 65 patients diagnosed with exudative neovascular AMD with type 1 MNV were recruited. Among the 65 included eyes, 35 eyes had no evidence or history of diabetes and were assigned to the non-DM group and 30 eyes had nonproliferative DR (NPDR) and were assigned to the DR group. None of the included eyes had received any prior treatment and were scheduled to undergo a loading dose of anti-VEGF therapy, which consisted of three monthly injections. The definition of mild NPDR was based on the presence of at least one microaneurysm and/or mild hemorrhages, using the modified Early Treatment Diabetic Retinopathy Study retinopathy severity scale.^23^

Exclusion criteria were applied to ensure the homogeneity of the study population and the reliability of the results. The criteria included (i) myopia greater than 3.00 diopters, (ii) infection or inflammation affecting both eyes, (iii) presence of type 2 or type 3 MNV, (iv) previous retinal treatment, (v) coexisting macular diseases, (vi) history of anti-VEGF injection or retinal laser therapy in the study eye, (vii) history of myocardial infarction or cerebrovascular disease within the last 6 months, (viii) significant cataract, (ix) any optic neuropathy such as glaucoma, and (x) neurodegenerative diseases like Alzheimer's disease or Parkinson's disease. Additionally, poor-quality images with incorrect segmentation or motion artifacts were excluded from the analysis.^24^

### Study Protocol

The study protocol involved conducting a comprehensive ophthalmologic examination at baseline, including assessment of best-corrected visual acuity (BCVA), IOP, and dilated ophthalmoscopy. Additionally, all patients underwent imaging with XR Avanti AngioVue OCTA (Optovue Inc, Fremont, CA). The study visits included the T1 visit, conducted the day before the first anti-VEGF injection, and the T2 visit, performed 1 month after the last injection in the loading phase. All patients received monthly aflibercept injections for a total of three injections. At each follow-up visit, a complete ophthalmologic evaluation was conducted to assess changes in BCVA, and OCT and OCTA imaging were performed to evaluate quantitative changes.

### Imaging Acquisition

Imaging acquisition was performed using XR Avanti AngioVue OCTA with 3 × 3-mm volume scans at a resolution of 500 pixels × 500 pixels. The Motion Correction Technology software was applied to correct image distortion in all directions. Scans with low quality owing to significant motion artifacts were excluded and repeated to achieve a signal quality of at least 8 out of 10.

The semiautomated segmentation algorithm provided by the manufacturer was used to delineate different retinal and choroidal layers. Specifically, the focus was on the OCTA en face flow image of the CC using a slab 10 µm thick, starting 31 µm posterior to the RPE–Bruch's membrane complex.^10^^,^^25^ Prior to image processing, two retinal specialists (PV and EB) independently reviewed each OCTA scan to ensure accurate segmentation and avoid potential errors.

**Figure 1.**
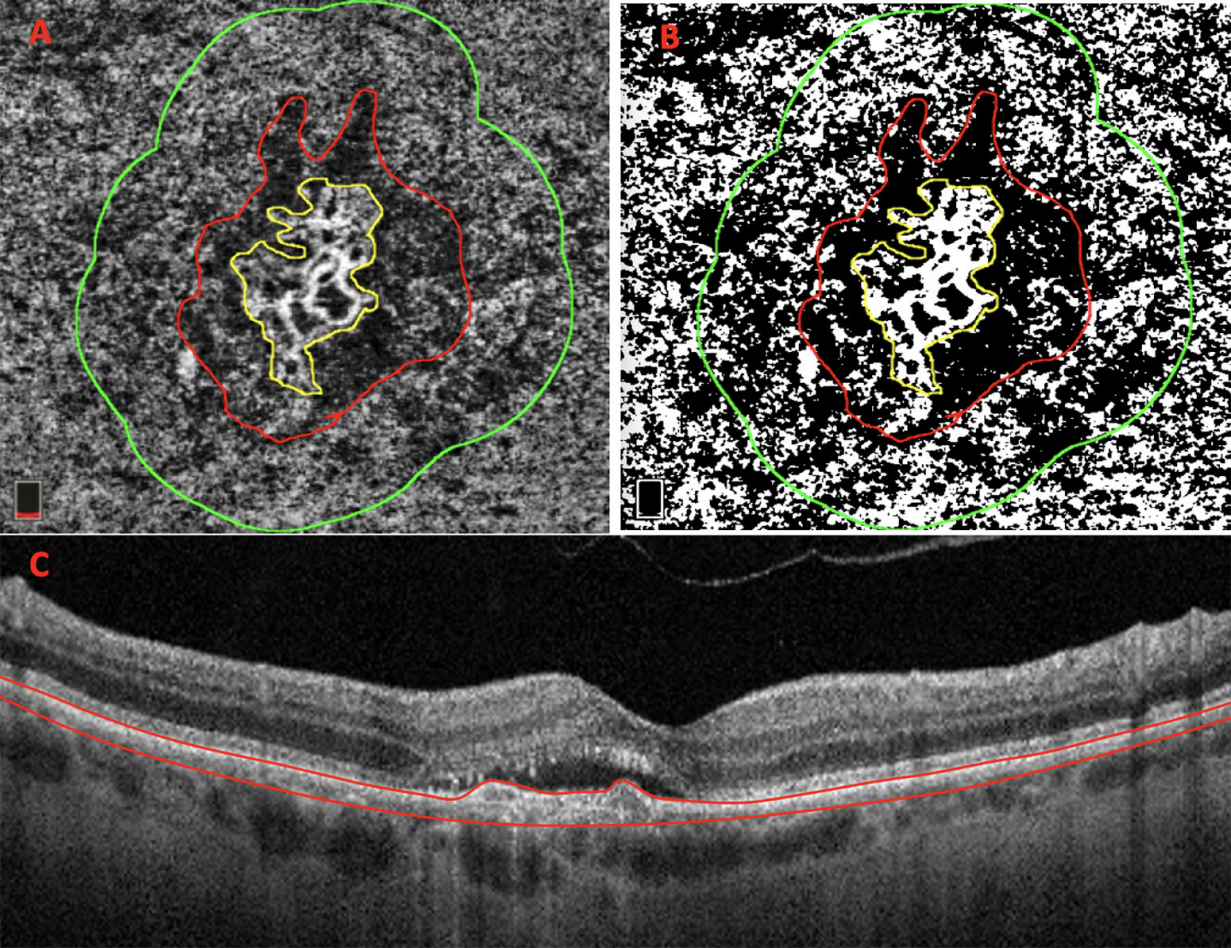
The 3 × 3-mm en face CC exhibits subfoveal treatment-naïve MNV. (**A**) En face CC OCTA images are used to manually delineate the borders of the MNV lesion (highlighted in *yellow*) and the related DH (highlighted in red). Afterward, we generated from the DH edge a 500-µm-wide ring region using the “Distance Map” function in ImageJ, which automatically creates a border that follows the contour of the perilesional halo (highlighted in *green*) (**B**) Then, the resulting CC images were binarized for quantitative measurement of the FD in the final ring using the Phansalkar method. (**C**) Structural OCT B-scan displays the corresponding CC segmentation. CC analysis was performed by analyzing OCTA en face (slab 30 µm thick starting 31 µm posterior to the RPE–Bruch's membrane complex).

CC areas beneath major superficial retinal vessels were excluded from the analysis to prevent possible shadows or projection artifacts^26^^,^^27^ Additionally, all MNV edges included in the analysis were required to be at least 1 mm away from the scan edge (Fig. 1).

### Image Processing

CC OCTA slabs were imported in Fiji ImageJ (software version 2.0.0; National Institute of Health, Bethesda, MD; available at http://rsb.info.nih.gov/ij/index.html). Using a previous algorithm proposed by Zhang et al.,^28^ CC images were compensated to remove additional shadowing or projection artifacts.

**Figure 2.**
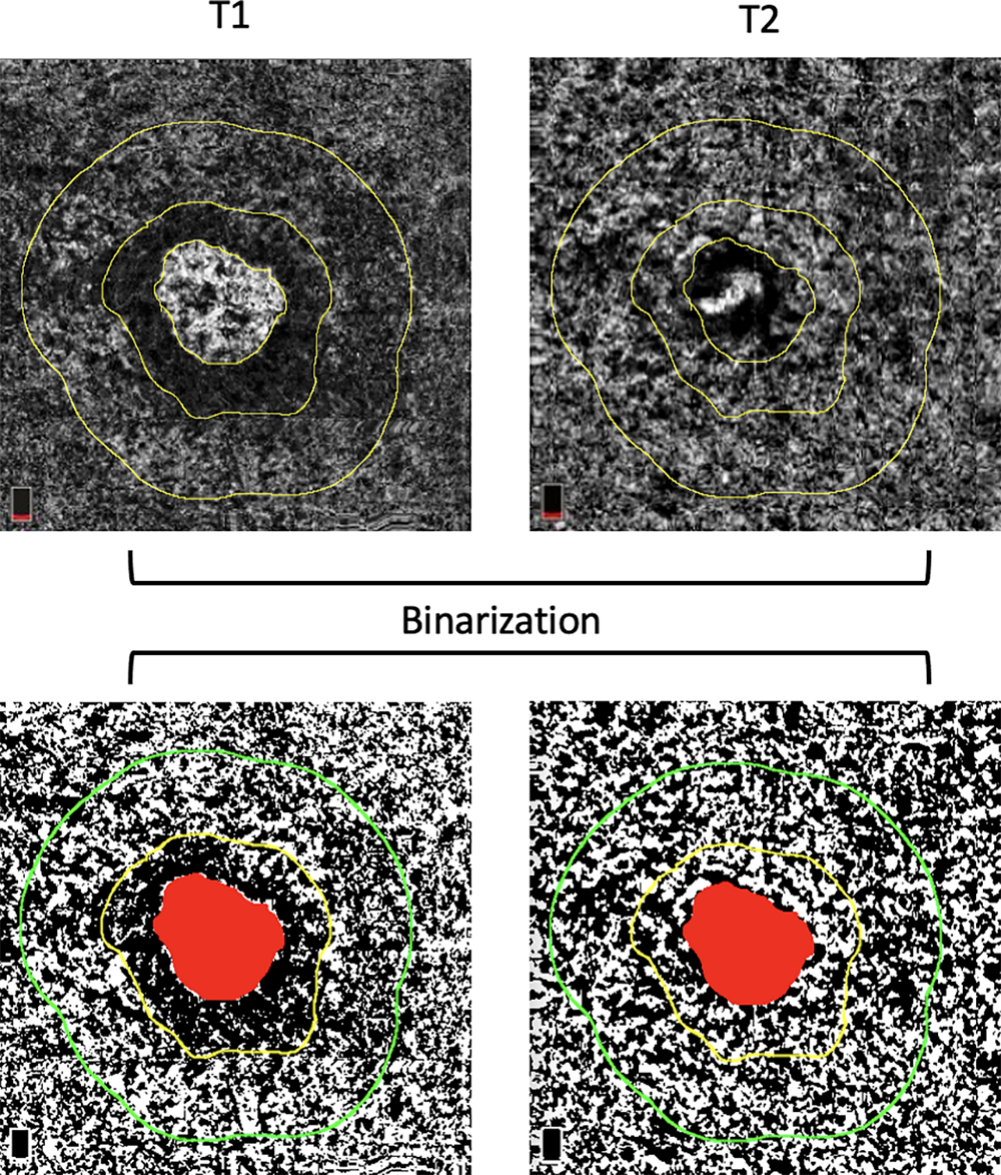
En face CC OCTA images show the borders of the MNV lesion, the related DH and the automated final ring (highlighted in *yellow*). Afterward, the CC images were binarized for quantitative FDs measurements in the final ring using the Phansalkar method at baseline (T1) and after loading dose of aflibercept (T2). The custom configuration, which is unique for each patient, was applied to the en face CC images at Time 2 using the same size and position of the rings as in T1.

Two masked expert graders (PV and EB) manually delineated the MNV borders and the related dark halo (DH) in each CC slab. Continuing, we generated from the DH edge a 500-µm-wide ring region using the “Distance Map” function in ImageJ, which automatically creates a border that follows the contour of the perilesional halo^29^ (Fig. 1). The final ring (500 µm ) was added to the region of interest manager for CC flow analysis. The custom configuration (unique for each patient) consisting of this ring was applied to the CC slab at T2 at the same size and position (Fig. 2).^10^

Afterward, using the Phansalkar method (radius of 15 pixels), CC slabs were binarized to obtain quantitative measurement of the FDs in the final ring.^9^^,^^30^^–^^32^ CC FDs were calculated in the final ring using the “analyze particles” command provided by ImageJ. In detail, the metrics included in the analysis were (i) the FD percentage (FD%), which represents the percentage of FDs within the analyzed area (FD%); (ii) the FD average area (FDa), which represents the average size of the FD within the analyzed region; and (iii) the FD number, which quantifies the number of FDs in the region of interest.

### Statistical Analysis

Statistical calculations were conducted using the Statistical Package for Social Sciences (SPSS IBM Statistic 25, Chicago, IL). The data distribution was assessed using the Shapiro–Wilk test. Paired *t*-tests were used for analyzing quantitative data before and after the loading phase injections in both groups. The Friedman nonparametric test was applied to compare delta changes between groups. A significance level of *P* < 0.05 was chosen to determine statistical significance.

## Results

### Characteristics of Patients Included in the Analysis

A total of 65 eyes of 65 patients with treatment-naïve neovascular AMD-associated type 1 MNV were included in the study. Forty patients were women, and twenty-five patients were men. The mean ± SD age of DM group was 78.4 ± 7.3 years (range, 56–87 years). The mean ± SD age of the non-DM group was 77.7 ± 6.1 years (range, 56–84 years). The comparison between two groups did not display significantly differences in terms of age (*P* ≥ 0.05). The characteristics of subjects included in the analysis are summarized in Table 1.

**Table 1.** Clinical Characteristic of Patients Included in the Analysis

Variables	DM Group	Non-DM Group	*P* Value
No.	30	35	>0.05
Age (years)	78.4 ± 7.3	77.7 ± 6.1	>0.05
Gender (female)	16 (53)	24 (68)	>0.05
Cardiovascular disease^*^	12 (40)	11 (31)	>0.05

Data are presented as mean ± SD or number (%).

* History of heart attack or stroke.

**Table 2.** Morphofunctional Results: Data and Comparisons (T1 vs T2)

	DM Group			Non-DM Group		
	T1	T2	*P* Value	T1	T2	*P* Value
BCVA (logMAR)	0.52 ± 0.17	0.38 ± 0.21	0.036	0.50 ± 0.19	0.35 ± 0.13	0.022
MNV lesion area (mm^2^)	1.01 ± 0.92	0.81 ± 0.74	0.032	0.98 ± 0.74	0.86 ± 0.73	0.018
DH (mm^2^)	1.99. ± 1.76	1.74 ± 1.49	0.039	2.02 ± 1.84	1.54 ± 1.37	0.042

Data are presented as mean ± SD.

T1, before loading anti-VEGF therapy; T2, after loading anti-VEGF therapy.

Concerning BCVA, it is noteworthy that both study groups exhibited a substantial improvement after the initial aflibercept loading dose. In the DM group, the mean BCVA was 0.52 ± 0.17 logMAR at T1 and improved to 0.38 ± 0.21 logMAR at T2 (*P* = 0.036). Likewise, in the non-DM group, the mean BCVA showed improvement, measuring 0.50 ± 0.19 logMAR at T1 and 0.35 ± 0.13 logMAR at T2 (*P* = 0.022) (Table 2).

Additionally, both groups demonstrated a notable decrease in the size of MNV lesions and the associated perilesional DH following antiangiogenic therapy. In the DM group, the average MNV area decreased from 1.01 ± 0.92 mm^2^ at T1 to 0.81 ± 0.74 mm^2^ at T2 (*P* = 0.032). In the non-DM group, the mean MNV lesion area decreased from 0.98 ± 0.74 mm^2^ at T1 to 0.86 ± 0.73 mm^2^ at T2 (*P* = 0.018). The perilesional DH area in DM eyes decreased from 1.99 ± 1.76 mm^2^ at T1 to 1.74 ± 1.49 mm^2^ at T2 (*P* = 0.039), whereas in non-DM eyes, it decreased from 2.02 ± 1.84 mm^2^ at T1 to 1.54 ± 1.37 mm^2^ at T2 (*P* = 0.042) (Table 2). The intraclass correlation coefficient for the MNV area analysis was 0.94, and for the DH area quantification it was 0.95.

### OCTA Analysis of the CC

#### DM Group

In the DM group, the topographical analysis of the CC using OCTA did not show any statistically significant changes among the different study visits. Specifically, the CC ring (500 µm) around the DH did not display a significant reduction in FD% at T2 compared with T1 (at T1: median, 54.61% [IQR, 51.18%–59.79%]; AT t2, median, 54.04% [IQR, 49.71%–60.12%]; *P* = 0.897). Similarly, there were no statistically significant differences in the average size (at T1: median, 108.28 [IQR, 78.75–176.76]; at TS: median, 92.64 [IQR, 67.58–150.52]; *P* = 0.247) and FD number size (at T1: median, 261.0 [IQR, 172.9–492.3]; at T2: median, 288.8 [IQR, 199.0–419.7]; *P* = 0.983) (Table 3).

**Table 3.** Topographical CC Analysis (Ring = 500 µm): Data and Comparisons (T1 vs T2)

	DM Group			Non-DM Group		
	T1	T2	*P* Value	T1	T2	*P* Value
CC FD%	54.61 [51.18–59.79]	54.04 [49.71–60.12]	0.897	51.51 [41.14–58.07]	47.09 [40.15–54.41]	0.003
CC FDa	108.28 [78.75– 176.76]	92.64 [67.58–150.52]	0.247	78.05 [41.59–119.81]	67.92 [35.41–87.27]	0.008
CC FDn	261.0 [172.9–492]	288.8 [199.0–419.7]	0.983	536.0 [319.8–890.8]	738.0 [382.0–1000]	0.003

Data are presented as median [IQR].

FDa, FD average area; FDn, flow deficits number; T1, before loading anti-VEGF therapy; T2, after loading anti-VEGF therapy.

#### Non-DM Group

Otherwise, in the non-DM group, the topographical CC analysis showed statistically significant changes after the loading phase of aflibercept. The CC ring around the DH displayed a progressive reduction in FD% after antiangiogenic therapy, indicating gradual reperfusion of the CC (at T1: median, 51.51% [IQR, 41.14–58.07]; at T2: median, 47.09% [IQR, 40.15–54.41]; *P* = 0.003). Additionally, the CC ring (500 µm) showed a significant progressive contraction of the FD average size after aflibercept therapy (at T1: median, 78.05 [IQR, 41.59–119.81]; at T2: median, 67.92 [IQR, 35.41–87.27]; *P* = 0.008). However, there was a significant increase in FD number size after anti-VEGF LP treatment compared with T1 (at T1: median, 536.0 [IQR, 319.8–890.8]; at T2: median, 738.0 [IQR, 382.0–1000.0]; *P* = 0.003) (Table 3).

#### Comparison Between Groups

**Figure 3.**
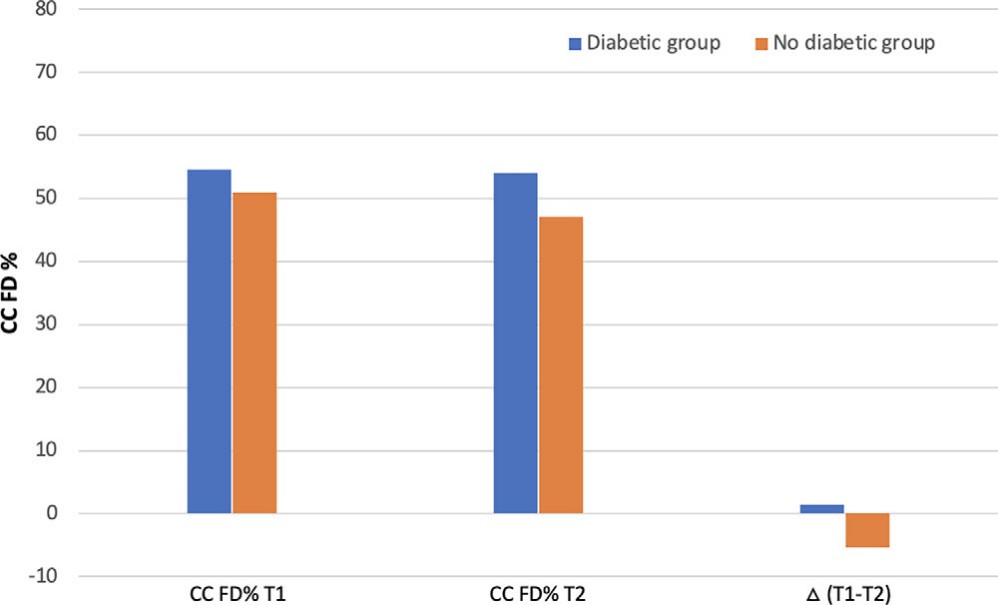
Sixty-five eyes with a diagnosis of exudative neovascular AMD and type 1 MNV were enrolled, resulting in a group of 35 subjects with no evidence or history of diabetes and in a group of 30 individuals with diabetes and mild NPDR. Results shown represent the Phansalkar local thresholding method analysis. As illustrated in the graph, the percentage (%) of CC FD is statistically significantly greater in the DM group compared with non-DM group in both T1 (*P* = 0.037) and T2 (*P =* 0.002). Also, the mean difference of CC FD% between the two groups is statistically significantly. A gradual CC reperfusion resulted in the “non-DM group” after LP of aflibercept compared with DM eyes (*P* = 0.029).

The comparison between the two groups revealed significant changes in the topographical CC analysis. Before the aflibercept intravitreal injection, the DM group had significantly higher FD% and FD average size compared with the non-DM group (*P* = 0.037; *P* = 0.005), indicating more severe CC impairment. After anti-VEGF treatment, the DM group still exhibited greater CC ischemia (higher FD% and FD average size) compared with the non-DM group (*P* = 0.002; *P* = 0.001) (Table 4). Moreover, the delta FD% between groups were statistically significant, indicating a gradual reperfusion of the CC in the non-DM group after anti-VEGF LP of aflibercept compared with the DM eyes (*P* = 0.029) (Fig. 3 and Table 5)

**Table 4.** Comparison Between DM vs Non-DM Eyes

	DM Group (*n* = 30)	Non-DM Group (*n* = 35)	*P* Value
CC FD% (T1)	54.61 [51.18–59.79]	51.51 [41.14–58.07]	0.037
CC FD% (T2)	54.04 [49.71–60.12]	47.09 [40.15–54.41]	0.002
CC FDa (T1)	108.28 [78.75–176.76]	78.05 [41.59–119.81]	0.005
CC FDa (T2)	92.64 [67.58–150.52]	67.92 [35.41–87.27]	0.001
CC FDn (T1)	261.0 [172.9–492]	536.0 [319.8–890.8]	0.068
CC FDn (T2)	288.8 [199.0–419.7]	738.0 [382.0–1000]	0.056

Data are presented as median [IQR].

FDa, FD average area; FDn, flow deficits number; T1, before loading anti-VEGF therapy; T2, after loading anti-VEGF therapy.

The Friedman nonparametric test was performed to obtain *P* values.

**Table 5.** Comparison Between DM and non-DM Eyes

	DM Group (*n* = 30)	Non-DM Group (*n* = 35)	*P* Value
Δ CC FD%	1.39 [−5.29 to 5.27]	−5.4 [−12.4 to −1.0]	0.029
Δ CC FDa	−5.97 [−33.9 to 23.3]	−17.8 [−31.8 to −2.4]	0.078
Δ CC FDn	7.13 [−19.0 to 27.9]	19.4 [5.6 to 39.0]	0.082

Data are delta percentages (median [IQR]).

FDa, FD average area; FDn, flow deficits number; T1, before loading anti-VEGF therapy; T2, after loading anti-VEGF therapy.

The Friedman nonparametric test was performed to obtain *P* values.

## Discussion

In this prospective OCTA study, we investigated the impact of DR on CC perfusion in eyes with type 1 MNV in the setting of neovascular AMD before and after the loading phase of anti-VEGF treatment. The study used OCTA to assess the CC remodeling (and differences thereof) after the loading phase of antiangiogenic therapy in eyes with neovascular AMD without DM versus eyes with neovascular AMD and mild NPDR.

Historically, CC examinations in DM eyes were restricted to post mortem histological research.^33^ The presence of fenestrated vessels did not allow correct visualization by classic retinal imaging such as indocyanine green angiography or fluorescein angiography.^34^ High-resolution histopathological studies demonstrated widened intercapillary space, narrowed vascular lumen, and massive CC dropouts in NPDR post mortem eyes.^35^ Successive OCTA studies further confirmed these angiopathic alterations. Nesper et al.^36^ calculated the nonperfusion CC area in healthy and DR eyes. The authors have found a significant CC hypoperfusion increase in DM eyes with noninvasive SD-OCTA. Similarly, in a study of 1222 eyes with 1 year of follow-up, Wang et al.^37^ measured the FD% in the macular region in patients with different DR severity stages. The authors have found that each 1% increase in baseline CC FD% was significantly associated with a 1.69 times for DR progression, suggesting the FD% as a novel biomarker for predicting the onset and progression of DR. Taking these results together, DM and DR predispose to morphological impairment of the choroidal layer.

Furthermore, impairment of the CC in intermediate and late AMD stages has been reported.^38^ In a cohort study of 42 eyes, Borrelli et al.^39^ investigated the CC flow in patients with iAMD compared with healthy controls. iAMD eyes displayed an increased hypoperfusion compared with control eyes, suggesting CC measures as a possible parameter for predicting the development of exudative AMD. In line with these results, Corvi et al.^40^^,^^41^ performed topographical analysis of the CC in 90 consecutive iAMD eyes with 12 months of follow-up. The authors demonstrated that CC hypoperfusion in the macula center was significantly and independently associated with MNV development.

Friedman postulated that the primary cause of AMD can be largely attributed to impaired choroidal perfusion.^42^ Recent advancements in OCTA have provided confirmation that the CC circulation is most compromised in the area surrounding MNV. However, achieving reliable quantitative results necessitates signal compensation and image averaging, as shown in several studies^14^^,^^15^ Treister et al.,^16^ for instance, used a 200-µm-ring termed the halo zone and revealed a significant increase in CC nonperfusion adjacent to all MNV lesions. In a prospective case series involving 80 eyes, Coscas et al.^43^ associated the presence of a DH with the obscuring effect caused by blood, intraretinal, or subretinal fluids, considering it a sign of active choroidal neovascularization requiring treatment. Nevertheless, it remains uncertain whether this DH truly signifies CC ischemia or is merely a shadow effect. To address this ambiguity, we examined CC flow directly outside of the DH, excluding the halo itself. Definitively, CC layer plays a crucial role in predicting the onset and progression of both AMD and DR.

Our findings revealed that the DM group exhibited greater hypoperfusion in the CC surrounding the type 1 neovascular membrane and associated DH compared with the non-DM group. This difference was observed both before the start of therapy and after the loading phase. These findings are important because OCTA-based metrics are currently used for managing neovascular AMD and DR.

Interestingly, our results proved that the non-DM group was characterized by a significant CC reperfusion in the area surrounding the DH after antiangiogenic therapy. CC FD% and FD average area were visibly reduced in eyes with type 1 MNV and AMD at T2. Assuming that type 1 MNV recruits the surrounding vascular system to grow, our results demonstrated blood CC flow sequestering in the area surrounding the DH before anti-VEGF treatment, followed by partial CC flow recovery when choroidal neovascularization activity decreases after treatment. In addition to observing a decrease in CC FD average area and CC FD%, our research also revealed an increase in the FD number after the initial loading phase of treatment. Taken together, these findings imply that a diminished MNV flow signal after intravitreal therapy is linked to the presence of smaller FDs that subsequently divide, leading to a higher overall count of FDs but a lower FD density. These findings confirmed our previous data on CC modifications before and after antiangiogenic therapy.^10^

Nevertheless, we did not find the same results in the DM group. On the contrary, this group was characterized by a higher level of CC ischemia. The amount of FD% and FD average size was significantly greater in DM group compared with non-DM group, already at T1. The latter follows a previous meta-analysis by Choi et al.,^44^ the authors suggested that DM angiopathy represents a risk factor for AMD development, stronger for late AMD than earlier stages. In addition, DM group was not characterized by relevant CC modifications after antiangiogenic treatment. None of the CC parameters analyzed displayed profound modifications.

Interestingly, the comparison between the two groups showed a significant difference in terms of percentage CC FD%. Together, these results suggest a discrepancy in treatment response between the two groups. We might hypothesize that the presence of DR in eyes affected by neovascular AMD might lead to higher levels of VEGF and, therefore, a greater CC hypoperfusion not followed by recovery of the CC flow after intravitreal treatment. The DR-related choroidal ischemia seems contribute to this mechanism, resulting in a significant lack of response to anti-VEGF therapy compared with non-DM group.

Last, but not least, it is important to emphasize that both retinal disorders could coexist, causing different trends both at baseline and during treatment response. The presence of DR in eyes affected by neovascular AMD could be negatively impacted. However, to the best of our knowledge, no study focused on the CC flow modifications before and after VEGF antagonist treatment in eyes affected by neovascular AMD and mild NPDR.

Based on our OCTA findings, we support the idea that DM eyes with neovascular AMD should be given more attention because they may require more proactive anti-VEGF treatment compared with patients affected by only neovascular AMD. Further studies based on a functional and not only morphological analysis are necessary to better understand the management of these patients. Using OCTA, this study is the first to perform a topographic CC flow analysis in AMD-associated MNV type 1 before and after loading anti-VEGF treatment.

The present OCTA study has limitations to consider when interpreting our findings. Our sample size was relatively small. Furthermore, our OCTA study lacks a control group, implying that differences during the follow-up could have been occurred even if the antiangiogenic therapy was not applied. The major limitation is that we analyzed CC images using spectral domain OCTA, which uses shorter wavelength light in comparison with swept source OCT angiography, resulting in less signal passing through the RPE. The latter limitation could influence the qualitative evaluation of en face OCTA, although conducted by two expert masked graders.

In conclusion, we provide the first fully integrated study of the DR impact on morphological CC modifications in AMD-associated type 1 MNV undergoing 3 monthly aflibercept intravitreal injections. Using OCTA, we showed a different response to intravitreal therapy in terms of CC flow in the DM group compared with the non-DM group. In particular, DM eyes were characterized by greater CC hypoperfusion already before starting treatment, then showing insignificant changes after intravitreal therapy compared with non-DM eyes. The latter feature indicates that DM is risk factor to consider in patients affected by neovascular AMD during antiangiogenic treatment. These results provide evidence that the DR impact might play a fundamental role not only in the development and progression of late AMD, but also in evaluating responses to antiangiogenic therapy. Future larger studies using advanced OCTA technology and longer follow-up are warranted to further investigate these findings.
